# Prevalence of alcohol use disorder among the Latvian general population and associations with the PHQ-9 screening results and sociodemographic factors

**DOI:** 10.1192/j.eurpsy.2022.619

**Published:** 2022-09-01

**Authors:** V.V. Vinogradova, A. Kivite-Urtane, J. Vrublevska, E. Rancans

**Affiliations:** 1 Riga Stradins University, Department Of Psychiatry And Narcology, Riga, Latvia; 2 Riga Stradins University, Department Of Public Health And Epidemiology, Riga, Latvia

**Keywords:** Depression, Alcohol use disorder, Epidemiology

## Abstract

**Introduction:**

Both alcohol use disorder (AUD) and depression are potentially disabling and economically burdensome disorders. There is no available information about the prevalence of alcohol use disorder in the general population of Latvia.

**Objectives:**

To determine the 12-month prevalence of AUD and the association with depressive symptoms and socio-demographic factors among the Latvian general population.

**Methods:**

Computer assisted face-to-face interviews were carried out in 2019-2020 among a representative sample of the Latvian adult population (n=2687). The study sample was selected using a stratifyed random sampling method. The respondents were interviewed using the Patient Health Questionnaire-9 (a score of ≥10 was defined as indicating the presence of a clinically relevant depressive symptoms) and the Mini-International Neuropsychiatric Interview. Descriptive statistics and Chi-square test was applied.

**Results:**

There were 1238 males (46.1%) and 1449 females (53.9%) recruited. Mean age of respondents was 49.9 (SD 18.2). The 12-month prevalence of AUD according to the M.I.N.I was 13.1% with a statistically significant difference between the genders: 23.6% in men and 4.1% in women (p<0.001). AUD was significantly more prevalent among the respondents younger than 40 years: 18.0% (p<0.001), especially among young (18-44 y.o.) men – 28.1% of all diagnosed cases (p<0.001); There was found an association between the severity stage of AUD and PHQ-9 screening results: 18.8% of those diagnosed with severe AUD had a score greater than 10 points in the PHQ-9 (p<0.001).

**Conclusions:**

Young men are at particularly high risk of alcohol use disorder. Those with diagnosed alcohol use disorder should be also screened for the depressive symptoms.

**Disclosure:**

No significant relationships.

